# Supplementary material for: VAMP2 regulates phase separation of α-synuclein
Source: Nat Cell Biol. 2024 Jul 1;26(8):1296–308. doi: 10.1038/s41556-024-01451-6 (PMC11322000; doi:10.1038/s41556-024-01451-6)
Supplement: Supplementary file 1 — Reporting Summary [file 41556_2024_1451_MOESM1_ESM.pdf]

nature portfolio

Corresponding author(s): Dr Janin Lautenschläger

Last updated by author(s): Apr 25, 2024

Reporting Summary

Nature Portfolio wishes to improve the reproducibility of the work that we publish. This form provides structure for consistency and transparency in reporting. For further information on Nature Portfolio policies, see our Editorial Policies and the Editorial Policy Checklist.

Statistics

For all statistical analyses, confirm that the following items are present in the figure legend, table legend, main text, or Methods section.

n/a	Confirmed	
☐	☒	The exact sample size (n) for each experimental group/condition, given as a discrete number and unit of measurement
☐	☒	A statement on whether measurements were taken from distinct samples or whether the same sample was measured repeatedly
☐	☒	The statistical test(s) used AND whether they are one- or two-sided<br>*Only common tests should be described solely by name; describe more complex techniques in the Methods section.*
☒	☐	A description of all covariates tested
☐	☒	A description of any assumptions or corrections, such as tests of normality and adjustment for multiple comparisons
☐	☒	A full description of the statistical parameters including central tendency (e.g. means) or other basic estimates (e.g. regression coefficient) AND variation (e.g. standard deviation) or associated estimates of uncertainty (e.g. confidence intervals)
☐	☒	For null hypothesis testing, the test statistic (e.g. F, t, r) with confidence intervals, effect sizes, degrees of freedom and P value noted<br>*Give P values as exact values whenever suitable.*
☒	☐	For Bayesian analysis, information on the choice of priors and Markov chain Monte Carlo settings
☒	☐	For hierarchical and complex designs, identification of the appropriate level for tests and full reporting of outcomes
☐	☒	Estimates of effect sizes (e.g. Cohen's d, Pearson's r), indicating how they were calculated

Our web collection on statistics for biologists contains articles on many of the points above.

Software and code

Policy information about availability of computer code

Data collection	Data collection was performed using Zen 2.3 (black edition) and Zen 2.6 (blue edition), IncuCyte 2021A, CLARIOStar 5.01 and ImageLab 6.0.1
Data analysis	Image analysis was performed using FIJI and Imaris 10.1.0, data analysis was performed using Excel 2016, OriginPro 2018, and Prism 9.3.1

For manuscripts utilizing custom algorithms or software that are central to the research but not yet described in published literature, software must be made available to editors and reviewers. We strongly encourage code deposition in a community repository (e.g. GitHub). See the Nature Portfolio guidelines for submitting code & software for further information.

Data

Policy information about availability of data

All manuscripts must include a data availability statement. This statement should provide the following information, where applicable:

- Accession codes, unique identifiers, or web links for publicly available datasets
- A description of any restrictions on data availability
- For clinical datasets or third party data, please ensure that the statement adheres to our policy

All Source data are provided with the study. NMR data have been deposited to BMRBdep (https://bmrb.io/deposit/) under the deposition number 52093, 52094, and 52095.

Research involving human participants, their data, or biological material

Policy information about studies with human participants or human data. See also policy information about sex, gender (identity/presentation), and sexual orientation and race, ethnicity and racism.

Reporting on sex and gender	N/A
Reporting on race, ethnicity, or other socially relevant groupings	N/A
Population characteristics	N/A
Recruitment	N/A
Ethics oversight	N/A

Note that full information on the approval of the study protocol must also be provided in the manuscript.

Field-specific reporting

Please select the one below that is the best fit for your research. If you are not sure, read the appropriate sections before making your selection.

☒ Life sciences ☐ Behavioural & social sciences ☐ Ecological, evolutionary & environmental sciences

For a reference copy of the document with all sections, see nature.com/documents/nr-reporting-summary-flat.pdf

Life sciences study design

All studies must disclose on these points even when the disclosure is negative.

Sample size	No statistical methods were used to pre-determine sample sizes but our sample sizes are similar to those reported in previous publications (Wu et al. 2021 Mol. Cell; Park et al. 2021 Nat. Commun; Park et al. 2023 Nat. Commun).
Data exclusions	Data were included if the control (wildtype) showed appropriate condensate formation.
Replication	All experiments have been replicated 2 or more times as indicated in the manuscript. All attempts of replication were successful.
Randomization	Samples were randomly allocated into experimental groups.
Blinding	Blinded analysis was performed for quantification of condensate formation (Incucyte experiments) and for quantitative analysis of condensates of the 96AAA aSYN variant. Image acquisition and analysis were performed non-blinded otherwise, but performed to an objectively defined standard and ensuring reproducibility.

Reporting for specific materials, systems and methods

We require information from authors about some types of materials, experimental systems and methods used in many studies. Here, indicate whether each material, system or method listed is relevant to your study. If you are not sure if a list item applies to your research, read the appropriate section before selecting a response.

Materials & experimental systems

n/a	Involved in the study	
☐	☒	Antibodies
☐	☒	Eukaryotic cell lines
☒	☐	Palaeontology and archaeology
☒	☐	Animals and other organisms
☒	☐	Clinical data
☒	☐	Dual use research of concern
☒	☐	Plants

Methods

n/a	Involved in the study	
☒	☐	ChIP-seq
☒	☐	Flow cytometry
☒	☐	MRI-based neuroimaging

Antibodies

Antibodies used	Anti-Flag Rabbit PolyAb (Proteintech, Cat No. 20543-1-AP, Lot 00098866)

Validation	Anti-Flag Rabbit PolyAb (Proteintech, Cat No. 20543-1-AP, Lot 00098866)<br><br>Manufacturer validation: HEK-293T cells and transfected HEK-293T lysates were subjected to SDS PAGE followed by western blot with 20543-1-AP (DYKDDDDK tag antibody) at dilution of 1:50000 incubated at room temperature for 1.5 hours. No band is seen for un-transfected HEK-293T cells. Western blot image is provided on the manufacturer website: https://www.ptglab.com/products/Flag-Tag-Antibody-20543-1-AP.htm<br><br>20543-1-AP targets DYKDDDDK tag in WB, IP, IF, FC, RIP, IHC, CoIP, ChIP, ELISA applications and shows reactivity with recombinant protein samples. Cited reactivity: Human, Mouse, Rat, Chicken, Yeast, Monkey, Pig, Duck, Immunogen: DYKDDDDK tag fusion protein Ag2329. RRID: AB_11232216

Eukaryotic cell lines

Policy information about cell lines and Sex and Gender in Research

Cell line source(s)	HeLa cells were obtained from the European Collection of Cell Cultures (ECACC 93021013). sex: female, human cell line
Authentication	Cell morphology
Mycoplasma contamination	Cells tested negative for mycoplasma contamination using the MycoStripTM assay (InvivoGen).
Commonly misidentified lines (See ICLAC register)	No commonly misidentified lines per the ICLAC register were used.
