# Supplementary material for: VAMP2 regulates phase separation of α-synuclein
Source: Nat Cell Biol. 2024 Jul 1;26(8):1296–308. doi: 10.1038/s41556-024-01451-6 (PMC11322000; doi:10.1038/s41556-024-01451-6)
Supplement: Supplementary file 12 — Unprocessed gels. [file 41556_2024_1451_MOESM12_ESM.pdf]

Source Data Fig. 4a/b

Coomassie gels for 4 biological repeats with analysis and bands marked for Csat calculation.

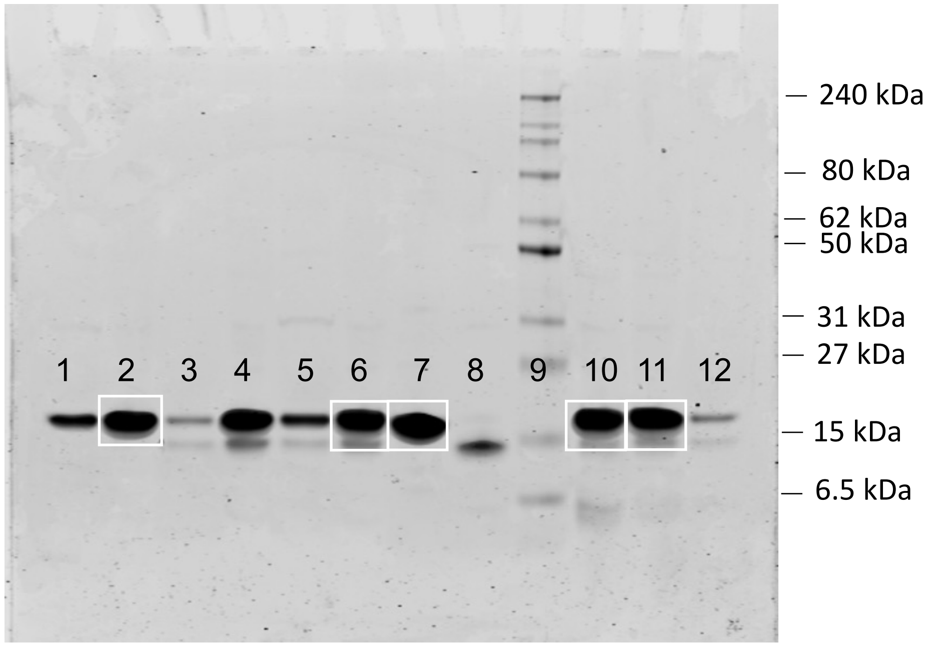

1) Synuclein (40 uM) LLPS: Pellet
2) Synuclein (40 uM) LLPS: Supernatant
3) Synuclein (40 uM) + VAMP 1 (10 uM)_No Ca: Pellet
4) Synuclein (40 uM) + VAMP 1 (10 uM)_No Ca: Supernatant
5) Synuclein (40 uM) + VAMP 1 (10 uM) + Ca: Pellet
6) Synuclein (40 uM) + VAMP 1 (10 uM) + Ca: Supernatant
7) Synuclein (40 uM)
8) VAMP 1 (10 uM)
9) Marker
10) Synuclein (40 uM) + VAMP 2 (10 uM) + Ca: Supernatant
11) Synuclein (40 uM) + VAMP 3 (10 uM) + Ca: Supernatant
12) Synuclein (40 uM) + VAMP 2 (10 uM) + Ca: Pellet

Syn	1	60577.23	23.308	1	**40**
Syn LLPS	2	45700.56	17.584	0.75	**30.18**
Syn + 15% PEG +VAMP 1	3	36286.45	13.961	0.6	23.96
Syn + 15% PEG + Ca+ VAMP 1	4	36608.33	14.085	0.6	**24.17**
Syn + 15% PEG + Ca+ VAMP 2	5	40560.99	15.606	0.67	**26.78**
Syn + 15% PEG + Ca+ VAMP 3	6	40170.33	15.456	0.66	**26.52**

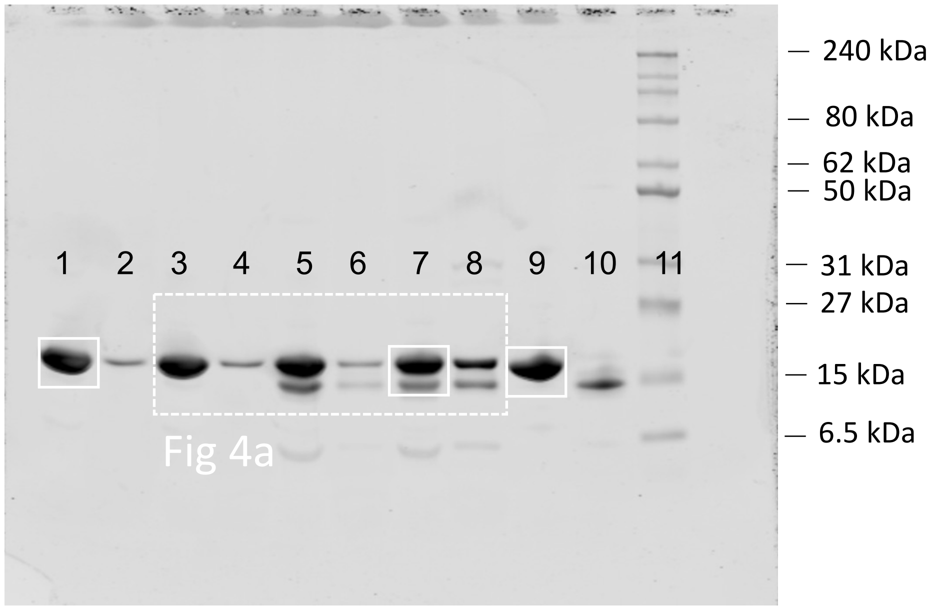

1) Synuclein (40 uM) LLPS: Sup
2) Synuclein (40 uM) LLPS: Pellet
3) Synuclein (40 uM) LLPS + NaCl: Sup
4) Synuclein (40 uM) LLPS + NaCl: Pellet
5) Synuclein (40 uM) + VAMP 1 (10 uM)_No Ca: Sup
6) Synuclein (40 uM) + VAMP 1 (10 uM)_No Ca: Pellet
7) Synuclein (40 uM) + VAMP 1 (10 uM) + Ca: Sup
8) Synuclein (40 uM) + VAMP 1 (10 uM) + Ca: Pellet
9) Synuclein (40 uM)
10) VAMP 1 (10 uM)
11) Marker

Syn	1	48126.4	23.332	1	40
Syn LLPS	2	45656.53	22.135	0.948697	37.94788
Syn LLPS +NaCl	3	43344.38	21.014	0.900651	**36.02606**
Syn + 15% PEG + VAMP	4	34652.47	16.8	0.720041	28.80165
Syn + 15% PEG + Ca+ VAMP	5	34486.77	16.72	0.716612	**28.6645**

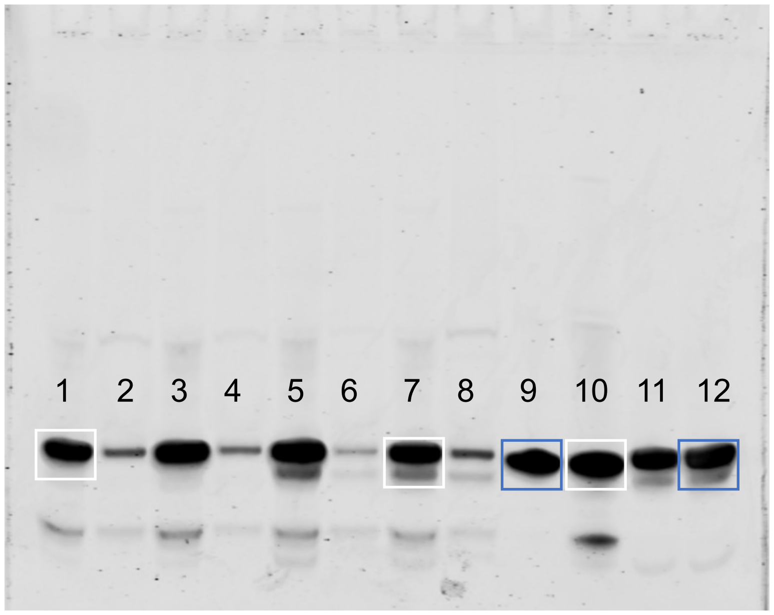

1) Synuclein (40 uM) LLPS: Sup (23_03)
2) Synuclein (40 uM) LLPS: Pellet
3) Synuclein (40 uM) LLPS + NaCl: Sup
4) Synuclein (40 uM) LLPS + NaCl: Pellet
5) Synuclein (40 uM) + VAMP 1 (10 uM)_No Ca: Sup
6) Synuclein (40 uM) + VAMP 1 (10 uM)_No Ca: Pellet
7) Synuclein (40 uM) + VAMP 1 (10 uM) + Ca: Sup
8) Synuclein (40 uM) + VAMP 1 (10 uM) + Ca: Pellet
9) Synuclein (40 uM)
10) Synuclein (40 uM)
11) Synuclein (40 uM) + VAMP 1 (10 uM)_No Ca: Pellet
12) Synuclein (40 uM) + VAMP 1 (10 uM) + Ca: Pellet

Syn	1	26333.23	24.807	1	**40**
Syn LLPS	2	20408.21	19.226	0.775023	31.00093
Syn LLPS +NaCl	3	26449.74	24.917	1.004434	**40.17737**
Syn + 15% PEG + VAMP	4	16646.44	15.682	0.63216	25.28641
Syn + 15% PEG + Ca+ VAMP	5	16313.77	15.368	0.619503	**24.7801**
Syn LLPS	1	25869.92	43.218	1	**40**
Syn + 15% PEG + VAMP	2	16928.28	28.28	0.654357	26.17428
Syn + 15% PEG + Ca+ VAMP	3	17061.03	28.502	0.659494	**26.37975**

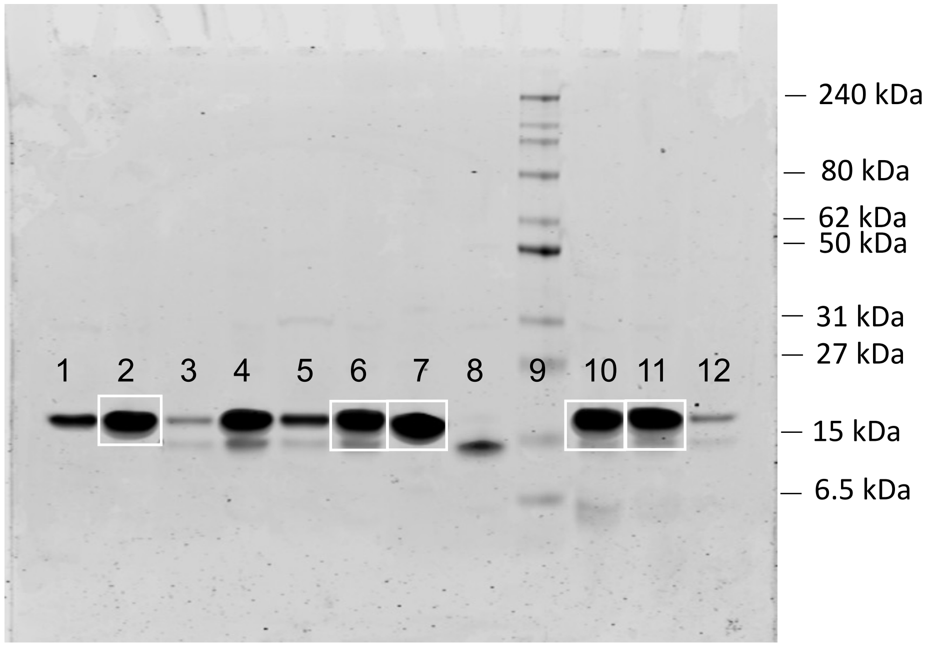

1) Synuclein (40 uM) LLPS: Pellet
2) Synuclein (40 uM) LLPS: Supernatant
3) **Synuclein (40 uM) + VAMP 1 (10 uM)_No Ca: Pelle**t
4) Synuclein (40 uM) + VAMP 1 (10 uM)_No Ca: Supernatant
5) **Synuclein (40 uM) + VAMP 1 (10 uM) + Ca: Pellet**
6) Synuclein (40 uM) + VAMP 1 (10 uM) + Ca: Supernatant
7) Synuclein (40 uM)
8) VAMP 1 (10 uM)
9) Marker
10) Synuclein (40 uM) + VAMP 2 (10 uM) + Ca: Supernatant
11) Synuclein (40 uM) + VAMP 3 (10 uM) + Ca: Supernatant
12) Synuclein (40 uM) + VAMP 2 (10 uM) + Ca: Pellet

	VAMP intensity
9621.501	42.313
13117.64	57.687

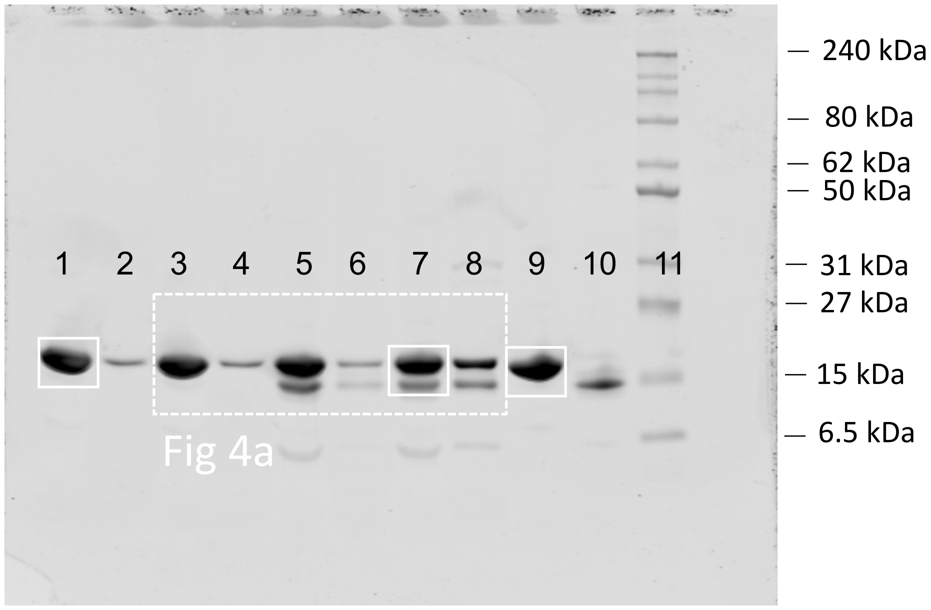

1) Synuclein (40 uM) LLPS: Sup
2) Synuclein (40 uM) LLPS: Pellet
3) Synuclein (40 uM) LLPS + NaCl: Sup
4) Synuclein (40 uM) LLPS + NaCl: Pellet
5) Synuclein (40 uM) + VAMP 1 (10 uM)_No Ca: Sup
6) **Synuclein (40 uM) + VAMP 1 (10 uM)_No Ca: Pellet**
7) Synuclein (40 uM) + VAMP 1 (10 uM) + Ca: Sup
8) **Synuclein (40 uM) + VAMP 1 (10 uM) + Ca: Pellet**
9) Synuclein (40 uM)
10) VAMP 1 (10 uM)
11) Marker

	VAMP intensity
1040.134	15.821
5534.196	84.179

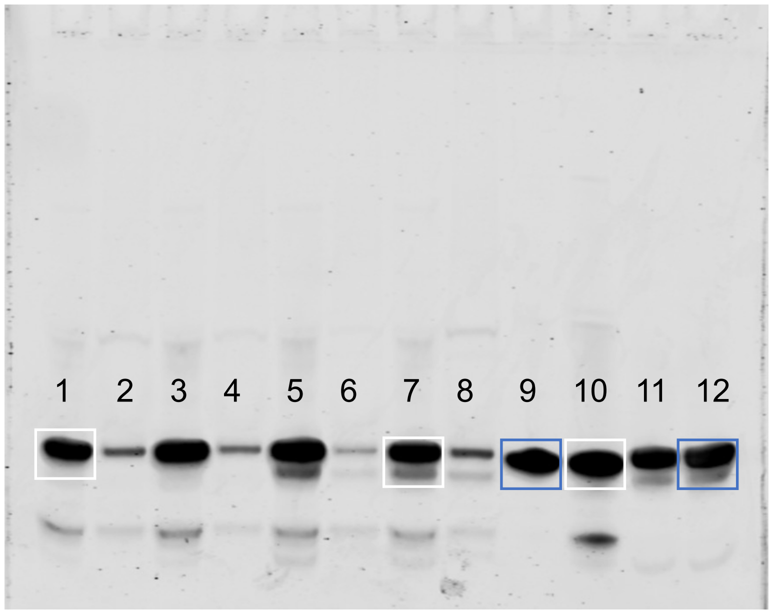

1) Synuclein (40 uM) LLPS: Sup (23_03)
2) Synuclein (40 uM) LLPS: Pellet
3) Synuclein (40 uM) LLPS + NaCl: Sup
4) Synuclein (40 uM) LLPS + NaCl: Pellet
5) Synuclein (40 uM) + VAMP 1 (10 uM)_No Ca: Sup
6) **Synuclein (40 uM) + VAMP 1 (10 uM)_No Ca: Pellet**
7) Synuclein (40 uM) + VAMP 1 (10 uM) + Ca: Sup
8) **Synuclein (40 uM) + VAMP 1 (10 uM) + Ca: Pellet**
9) Synuclein (40 uM) 09_03
10) Synuclein (40 uM) 23_03
11) Synuclein (40 uM) + VAMP 1 (10 uM)_No Ca: Pellet
12) Synuclein (40 uM) + VAMP 1 (10 uM) + Ca: Pellet

	VAMP intensity			VAMP intensity
3051.933	25.11	1	463.933	34.929
9102.329	74.89	2	864.276	65.071
