# Supplementary material for: VAMP2 regulates phase separation of α-synuclein
Source: Nat Cell Biol. 2024 Jul 1;26(8):1296–308. doi: 10.1038/s41556-024-01451-6 (PMC11322000; doi:10.1038/s41556-024-01451-6)
Supplement: Supplementary file 15 — Unprocessed gels. [file 41556_2024_1451_MOESM15_ESM.pdf]

Source Data Fig. 6a

a) Coomassie gels for 4 biological repeats. Bands used for Csat calculation are highlighted in bold.
b) Coomassie gels with respective analysis of the bands highlighted in a.

a

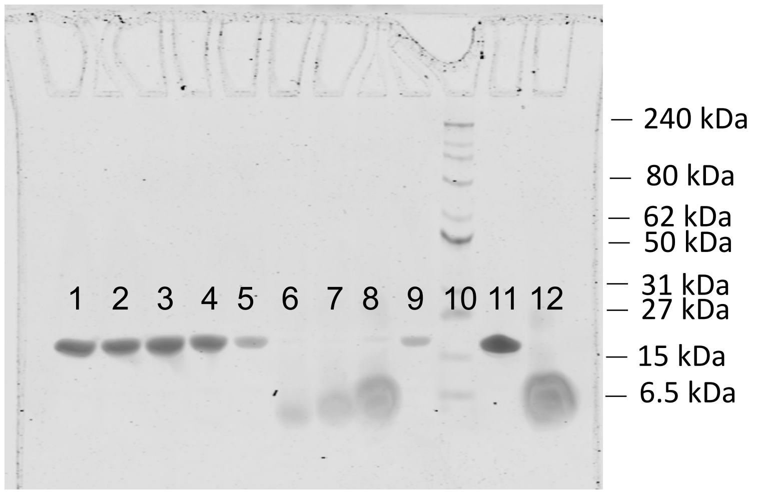

Supernatant

1) **Synuclein (40 uM) LLPS**
2) Synuclein (40 uM) + Peptide 5 long (1 uM)
3) **Synuclein (40 uM) + Peptide 5 long (10 uM)**
4) **Synuclein (40 uM) + Peptide 5 long (50 uM)**
5) **Synuclein (40 uM) + Peptide 5 long (100 uM)**
6) **Synuclein (40 uM) + Peptide 5 long (250 uM)**
7) Synuclein (40 uM) + Peptide 5 long (500 uM)
8) Synuclein (40 uM) + Peptide 5 long (1000 uM)
9) Synuclein (100 uM) LLPS + Peptide 5 long (100 uM)
10) Marker
11) Synuclein (40 uM)
12) Control Peptide + PEG

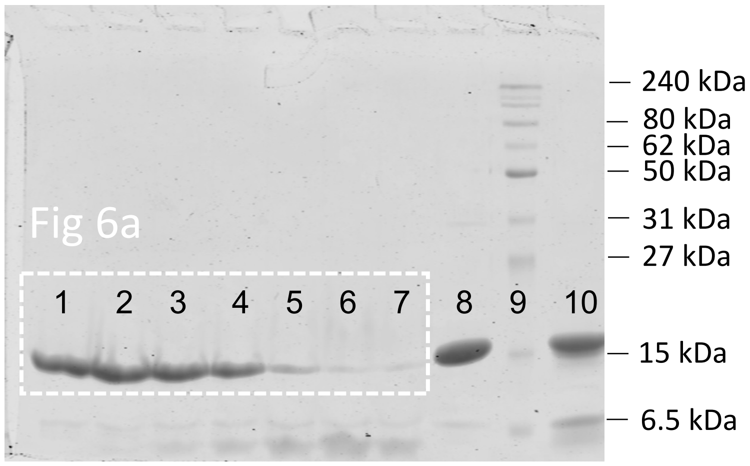

Supernatant

1) **Synuclein (40 uM) LLPS**
2) **Synuclein (40 uM) + Peptide 5 long (10 uM)**
3) **Synuclein (40 uM) + Peptide 5 long (50 uM)**
4) **Synuclein (40 uM) + Peptide 5 long (100 uM)**
5) **Synuclein (40 uM) + Peptide 5 long (150 uM)**
6) **Synuclein (40 uM) + Peptide 5 long (200 uM)**
7) **Synuclein (40 uM) + Peptide 5 long (250 uM)**
8) Synuclein (40 uM)
9) Marker
10) Synuclein (40 uM)

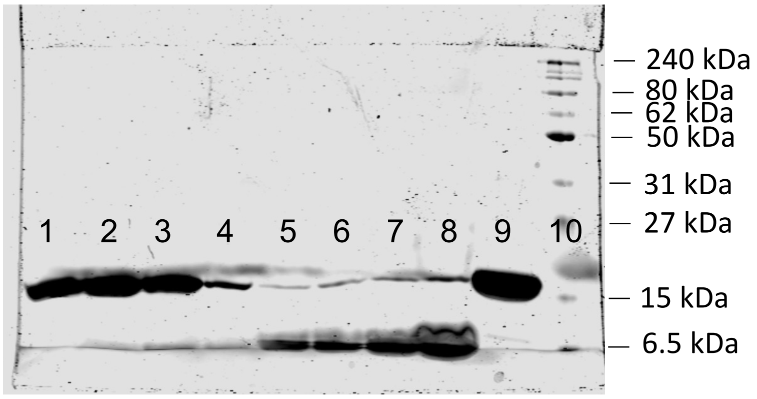

Supernatant

1) **Synuclein (40 uM) LLPS**
2) **Synuclein (40 uM) + Peptide 5 long (10 uM)**
3) **Synuclein (40 uM) + Peptide 5 long (50 uM)**
4) **Synuclein (40 uM) + Peptide 5 long (100 uM)**
5) **Synuclein (40 uM) + Peptide 5 long (150 uM)**
6) **Synuclein (40 uM) + Peptide 5 long (200 uM)**
7) **Synuclein (40 uM) + Peptide 5 long (250 uM)**
8) Synuclein (40 uM) + Peptide 5 long (500 uM)
9) Synuclein (40 uM)
10) Marker

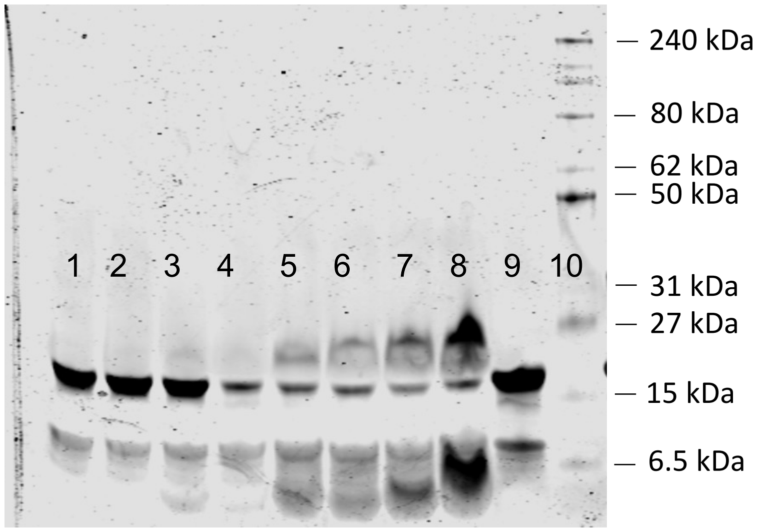

Supernatant

1) **Synuclein (40 uM) LLPS**
2) **Synuclein (40 uM) + Peptide 5 long (10 uM)**
3) **Synuclein (40 uM) + Peptide 5 long (50 uM)**
4) **Synuclein (40 uM) + Peptide 5 long (100 uM)**
5) **Synuclein (40 uM) + Peptide 5 long (150 uM)**
6) **Synuclein (40 uM) + Peptide 5 long (200 uM)**
7) **Synuclein (40 uM) + Peptide 5 long (250 uM)**
8) Synuclein (40 uM) + Peptide 5 long (500 uM)
9) Synuclein (40 uM)
10) Marker

a

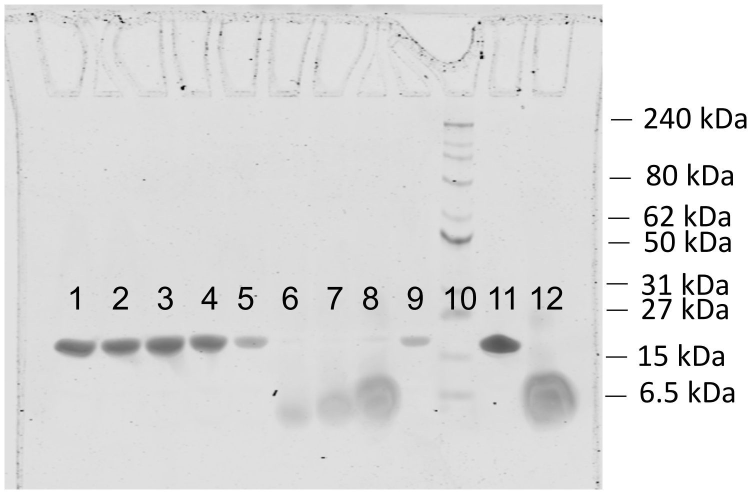

					Csat
	1	22335.92	22.35	1	40
**Synuclein (40 uM) LLPS**	**2**	**17344.77**	**17.356**	**0.776555**	**31.06219**
Peptide 5 long (1 uM)	3	16864.97	16.876	0.755078	30.20313
**Peptide 5 long (10 uM)**	**4**	**19993.23**	**20.006**	**0.895123**	**35.80492**
**Peptide 5 long (50 uM)**	**5**	**15202.89**	**15.213**	**0.680671**	**27.22685**
**Peptide 5 long (100 uM)**	**6**	**4744.468**	**4.748**	**0.212438**	**8.497539**
**Peptide 5 long (250 uM)**	**7**	**85.95**	**0.086**	**0.003848**	**0.153915**
Peptide 5 long (500 uM)	8	26.121	0.026	0.001163	0.046532
Peptide 5 long (1000 uM)	9	298.849	0.299	0.013378	0.535123
Synuclein (40 uM) LLPS + P5Long 100 uM	10	3038.569	3.041	0.136063	5.442506

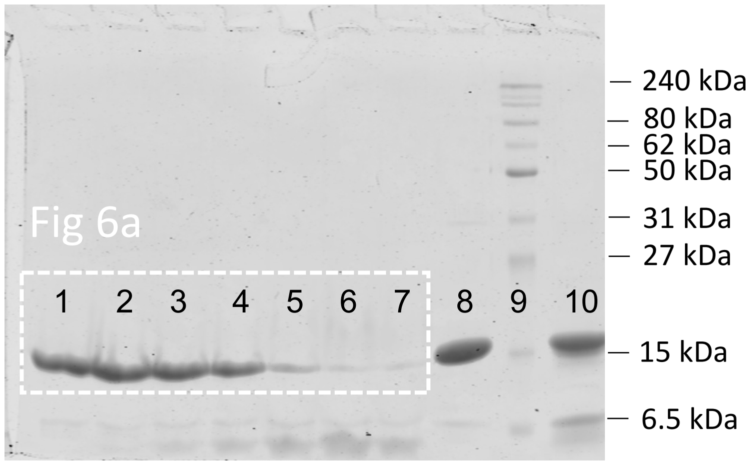

					Csat
	1	27586.25	22.94	1	40
**Synuclein (40 uM) LLPS**	**2**	**22873.32**	**19.021**	**0.829163**	**33.16652**
**Peptide 5 long (10 uM)**	**3**	**26568.27**	**22.094**	**0.963121**	**38.52485**
**Peptide 5 long (50 uM)**	**4**	**21976.35**	**18.275**	**0.796643**	**31.86574**
**Peptide 5 long (100 uM)**	**5**	**15120.55**	**12.574**	**0.548126**	**21.92502**
**Peptide 5 long (150 uM)**	**6**	**4257.761**	**3.541**	**0.154359**	**6.174368**
**Peptide 5 long (200 uM)**	**7**	**1130.477**	**0.94**	**0.040976**	**1.639058**
**Peptide 5 long (250 uM)**	**8**	**739.335**	**0.615**	**0.026809**	**1.072363**

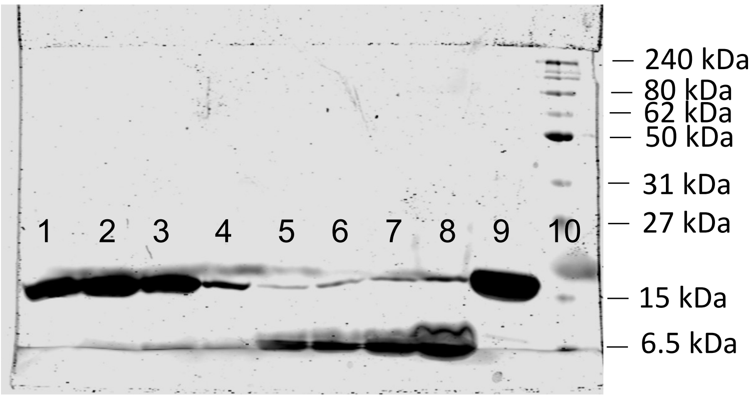

					Csat
	1	40201.64	25.524	1	40
**Synuclein (40 uM) LLPS**	**2**	**28262.96**	**17.944**	**0.703025**	**28.12098**
**Peptide 5 long (10 uM)**	**3**	**35811.26**	**22.737**	**0.890809**	**35.63235**
**Peptide 5 long (50 uM)**	**4**	**31659.35**	**20.101**	**0.787533**	**31.50133**
**Peptide 5 long (100 uM)**	**5**	**8141.409**	**5.169**	**0.202515**	**8.100611**
**Peptide 5 long (150 uM)**	**6**	**1693.598**	**1.075**	**0.042117**	**1.684689**
**Peptide 5 long (200 uM)**	**7**	**1766.255**	**1.121**	**0.043919**	**1.756778**
**Peptide 5 long (250 uM)**	**8**	**2195.054**	**1.394**	**0.054615**	**2.184611**
Peptide 5 long (500 uM)	9	7773.388	4.935	0.193347	7.733898

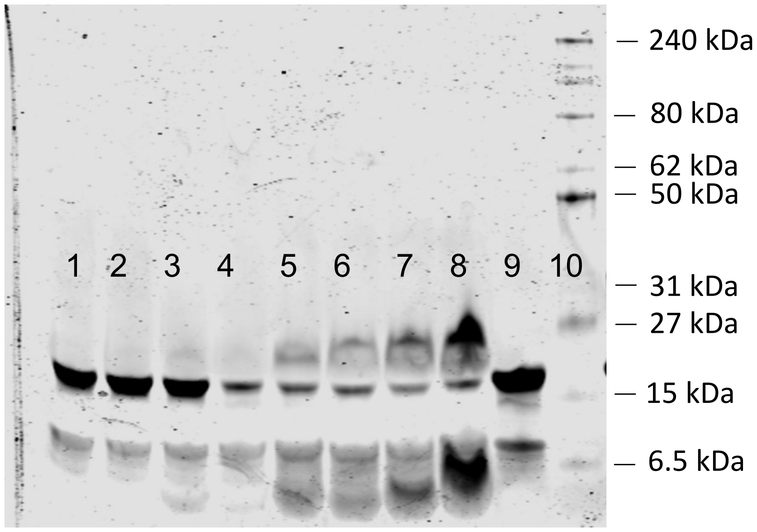

					**Csat**
	1	21094.3	21.804	1	40
**Synuclein (40 uM) LLPS**	**2**	**17614.5**	**18.207**	**0.83503**	**33.40121**
**Peptide 5 long (10 uM)**	**3**	**19375.79**	**20.027**	**0.918501**	**36.74005**
**Peptide 5 long (50 uM)**	**4**	**16151.75**	**16.695**	**0.765685**	**30.62741**
**Peptide 5 long (100 uM)**	**5**	**5734.589**	**5.927**	**0.271831**	**10.87323**
**Peptide 5 long (150 uM)**	**6**	**4618.054**	**4.773**	**0.218905**	**8.756192**
**Peptide 5 long (200 uM)**	**7**	**4981.347**	**5.149**	**0.236149**	**9.445973**
**Peptide 5 long (250 uM)**	**8**	**3360.518**	**3.474**	**0.159329**	**6.373143**
Peptide 5 long (500 uM)	9	3816.146	3.944	0.180884	7.23537
